# Sphingolipids as Potential Therapeutic Targets against Enveloped Human RNA Viruses

**DOI:** 10.3390/v11100912

**Published:** 2019-10-01

**Authors:** Eric J. Yager, Kouacou V. Konan

**Affiliations:** 1 Department of Basic and Clinical Sciences, Albany College of Pharmacy and Health Sciences, Albany, NY 12208, USA; Eric.Yager@acphs.edu; 2 Department of Immunology and Microbial Disease, Albany Medical College, Albany, NY 12208-3479, USA

**Keywords:** sphingolipids, glycosphingolipids, viruses, lipid biosynthesis, antiviral

## Abstract

Several notable human diseases are caused by enveloped RNA viruses: Influenza, AIDS, hepatitis C, dengue hemorrhagic fever, microcephaly, and Guillain–Barré Syndrome. Being enveloped, the life cycle of this group of viruses is critically dependent on host lipid biosynthesis. Viral binding and entry involve interactions between viral envelope glycoproteins and cellular receptors localized to lipid-rich regions of the plasma membrane. Subsequent infection by these viruses leads to reorganization of cellular membranes and lipid metabolism to support the production of new viral particles. Recent work has focused on defining the involvement of specific lipid classes in the entry, genome replication assembly, and viral particle formation of these viruses in hopes of identifying potential therapeutic targets for the treatment or prevention of disease. In this review, we will highlight the role of host sphingolipids in the lifecycle of several medically important enveloped RNA viruses.

## 1. Introduction

Human viruses come in various shapes and sizes with DNA or RNA as their genetic material. The focal point of this review is on enveloped RNA virus particles, which consist of a lipid bilayer typically surrounding the genomic-RNA-protecting shell or capsid. While the lipid composition of the envelope varies between these RNA viruses, it is often enriched in phospholipids, cholesterol, and sphingolipids. In many instances, the integrity of the virus envelope is crucial for viral infectivity. For example, specific phospholipids in the envelope of some members of the Flaviviridae family of viruses are reported to facilitate virus attachment to host cells, a key step in viral entry [1,2,3].

Enveloped RNA viruses are further divided into two classes based on the polarity of the genome. For example, most positive-stranded RNA viruses replicate exclusively in the cytoplasm of the infected cell and in intimate contact with intracellular membranes. This strategy enables viral and host factors to concentrate in distinct cellular locations to optimize a new virus particle’s formation and evade innate immune responses [4,5,6,7,8]. By contrast, the replication cycles of some negative-stranded RNA viruses (e.g. Influenza virus), and human immunodeficiency virus, occurs in the nucleus [9,10]. Hence, positive and negative-stranded RNA viruses require a distinct set of host membranes, and the lipids present therein, for successful virus propagation. This review will feature a few medically important enveloped RNA viruses, such as hepatitis C virus (HCV), dengue virus (DENV), Zika virus (ZIKV), human immunodeficiency virus (HIV), and influenza virus (IAV), and highlight the role of sphingolipids in their replication cycle and pathogenesis.

**Figure 1:**
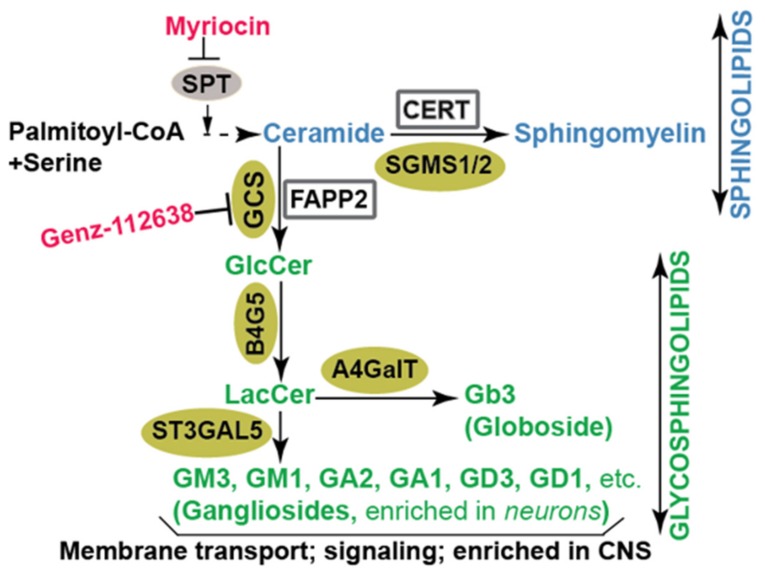
Diagram of sphingolipid biosynthetic pathways in mammalian cells. The initial step in the de novo biosynthesis of sphingolipids is the conversion of serine and palmitoyl CoA to ceramide. Following that, ceramide is subjected to conversion to sphingomyelin or to various glycosphingolipid intermediates on their way to becoming complex glycosphingolipids. The enzymes involved in the synthesis of sphingolipids and glycosphingolipids are denoted in gold. Chemical inhibitors of key enzymes are indicated in red. SGMS1/2: sphingomyelin synthase; GCS: glucosylceramide synthase; B4G5: lactosylceramide synthase; ST3GAL5: lactosylceramide alpha-2,3-sialyltransferase or GM3 synthase; A4GalT: alpha 1,4-galactosyltransferase or Gb3 synthase; SPT: serine palmitoyl transferase; CERT: ceramide transfer protein; FAPP2: four-phosphate adaptor protein 2; GlcCer: glucosylceramide; LacCer: lactosylceramide.

Sphingolipids are important biomolecules found in all eukaryotic membranes. They regulate membrane trafficking, cell signaling, and play a crucial role in influenza virus particles’ release or cell surface binding of HIV-1 glycoprotein gp120 [11,12]. They are also major constituents of lipid rafts, which are integral components of the HCV replication complex [13,14]. Sphingolipid biosynthesis starts with the conversion of palmitoyl-CoA and serine into ceramide, a sphingolipid byproduct of the endoplasmic reticulum (ER) resident enzyme—serine palmitoyltransferase, or SPT [15,16,17,18] (Figure 1). Ceramide can be carried by ceramide transport protein (CERT) [19] to the trans-Golgi where it is converted into another sphingolipid called sphingomyelin. Alternatively, four-phosphate adaptor protein 2, or FAPP2 [20], carries ceramide to the cis-Golgi where glucosylceramide synthase (GCS) produces the first glycosphingolipid called glucosylceramide (GlcCer; Figure 1). Glucosylceramide is subsequently converted into more complex glycosphingolipids including lactosylceramide (LacCer), globosides (e.g., Gb3) and gangliosides (e.g., GM3, GM1, and GA1; Figure 1). GCS is the rate-limiting enzyme in glycosphingolipid biosynthesis. The insufficiency or overproduction of glycosphingolipids has been associated with disease in humans. Consequently, efforts were made to inhibit GCS activity to reduce glucosylceramide accumulation in patients. One such GCS inhibitor, Genz-112638 [21], has been approved for treating Gaucher disease linked to defective glucosylceramide catabolism [22,23,24,25] (Figure 1). Sphingolipids and glycosphingolipids are found in distinct internal membranes as well as the plasma membrane. Additionally, glycosphingolipids are highly enriched in neurons, skin epithelial cells and might contribute to the tropism, replication and pathogenicity of viruses targeting related organs. Traditional methods to detect sphingolipids and glycosphingolipids include thin liquid chromatography (TLC) [20], high pressure thin liquid chromatography (HPTLC) [26], immunocytochemistry, and enzyme-linked immunosorbent assay (ELISA) [27,28]. Recently, sphingolipids and glycosphingolipids have been detected with state-of-the-art liquid chromatography coupled with mass spectrometry (e.g., LC-MS/MS system). This approach has enabled investigators to accurately determine the levels of sphingolipid and glycosphingolipid species in cells or tissues [28,29] and the impact of viral infection on levels of these lipids [30].

## 2. Hepatitis C Virus Propagation and Sphingolipids

**Figure 2:**
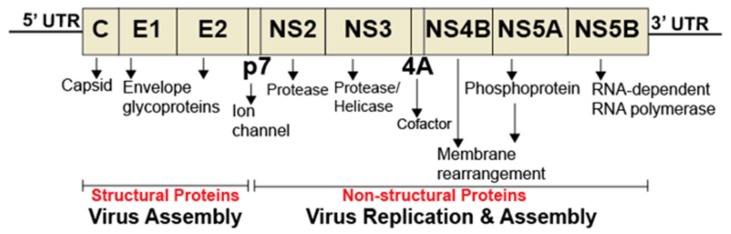
Diagram of hepatitis C virus genome. The HCV genome consists of a single open reading frame (ORF) flanked by the 5’ and 3’ untranslated regions (UTRs). The ORF is translated into a single polyprotein, which is further processed into individual proteins. The 5’ and 3’ UTRs are critical for internal ribosome binding, translation, and HCV genome replication.

Hepatitis C virus (HCV) is responsible for chronic liver disease in 60–90 million people worldwide [31] and is a member of the Flaviviridae family of viruses that encompass dengue virus, West Nile virus, and Zika virus. HCV is an enveloped virus with a positive-stranded RNA genome encoding structural proteins (Core, E1, and E2) (Figure 2) required for infectious HCV particles formation. The nonstructural proteins (NS2, NS3, NS4A, NS4B, NS5A, and NS5B) are involved in the replication of HCV genome and the packaging of the newly generated genome [32,33]. Major insights in the molecular and structural biology of HCV have led to the development of direct acting antivirals (DAAs) targeting HCV NS34/A protease (e.g., Simeprivir and Voxilaprevir), NS5A (e.g., Ledipasvir and Pibrentasvir), and NS5B RNA-dependent RNA polymerase or RDRp (e.g., Sofosbuvir and Dasabuvir) [34,35]. However, the high error rate (2.5 × 10^-5^ per nucleotide per genome replication) [36] of the HCV RdRp has resulted in the presence of resistance-associated variants [37,38]. Thus, novel antivirals targeting other HCV proteins, or host factors, are needed.

### 2.1. Sphingolipids and HCV Entry

There is strong evidence in the literature implying that sphingolipids and glycosphingolipids play an intimate role in HCV replication in liver cells. First, Merz et al. utilized lipid mass spectrometry to demonstrate that affinity-purified HCV particles are enriched in sphingomyelin [39], suggesting that sphingolipids are integral components of HCV envelope. This study did not address the role of sphingomyelin in HCV propagation, but another study by Aizaki et al. [40] demonstrated that sphingomyelin facilitates HCV internalization, perhaps via fusion of the virus envelope with endocytic membrane to release HCV genome into the cytoplasm.

### 2.2. Sphingolipids, Glycosphingolipids, and HCV Genome Replication

Sphingolipids are also known to facilitate HCV genome replication. In one study led by Hirata et al. [14], the authors showed that HCV infection stimulates sphingomyelin production, leading to sphingomyelin enrichment in the HCV replication complex and sphingomyelin-induced stimulation of HCV RNA-dependent RNA polymerase to synthesize more viral RNA. During infection, HCV induces the formation of a distinct membrane structure in the cell. This platform called the membranous web [4,41,42] recruits viral and host factors to foster HCV genome amplification. In another study from our laboratory, we found that HCV redirects the glycosphingolipid carrier protein, FAPP2 (Figure 1), to the membranous web to facilitate HCV genome replication [20]. It is likely that FAPP2 transports glycosphingolipids to the virus replication platform, as FAPP2 knockdown impedes HCV replication, whereas providing glycosphingolipids to the FAPP2 knockdown cells rescues HCV genome replication [20]. Finally, FAPP2 knockdown was found to disrupt the membranous web and alter the colocalization of HCV replicase proteins, implying that FAPP2 and/or glycosphingolipids contribute to the formation or maintenance of the HCV replication platform.

## 3. Flavivirus Propagation and Sphingolipids

**Figure 3:**
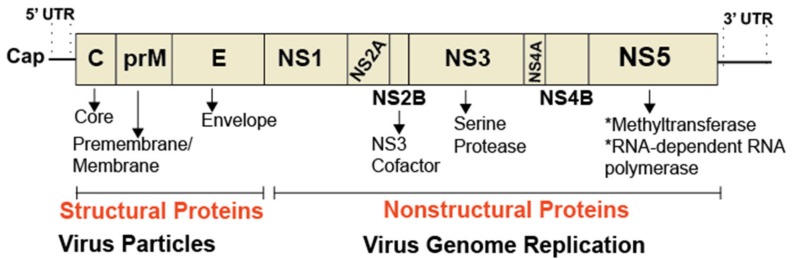
Organization of a flavivirus genome. The genome of flaviviruses, such as dengue and Zika, is ~11 kb in size and encodes a single, large polyprotein, which is proteolytically processed into three structural proteins and seven nonstructural proteins. The 5’ end of the genome contains a cap structure critical for the initiation of translation. RNA structures present in the 5’ and 3’ untranslated regions (UTR) are critical for capping and genome replication.

Dengue virus (DENV), West Nile virus (WNV), and Zika virus (ZIKV) are archetype flaviviruses transmitted by mosquitoes, mainly *Aedes aegypti* and *Aedes albopictus*. According to the world health organization, DENV is responsible for 50–100 million infections each year with mild complications resembling flu-like symptoms and major complications, including deadly dengue hemorrhagic fever. WNV causes flu-like symptoms, neuroinvasive disease, and death in many countries in the world. WNV was first introduced in the United States (US) in 1999 from infected Israeli birds imported into New York state. The virus has now spread to most states and is responsible for many deaths in birds, humans and horses. By contrast, ZIKV only emerged as a global health concern since 2016, due its association with neurological disorders, such as microcephaly in newborns and Guillain–Barré syndrome in adults [27,43]. Like hepatitis C virus (HCV), DENV, WNV, and ZIKV are enveloped viruses. Unlike HCV, the positive-stranded RNA genome of these flaviviruses encodes a slightly different set of structural proteins (Core, E, and prM) (Figure 3) required for virus particle formation, and nonstructural proteins (NS1, NS2A, NS2B, NS3, NS4A, NS4B, NS5) involved in viral genome replication, packaging, and pathogenesis [44,45,46]. No effective vaccine or specific antiviral treatments are currently available for DENV, WNV, or ZIKV infection.

### 3.1. DENV Propagation and Sphingolipids

There is evidence that sphingolipids and glycosphingolipids are also required for the replication of some flaviviruses. For example, DENV has been reported to upregulate the expression of sphingolipids (ceramide and sphingomyelin) in mosquito cells and cause an accumulation of these lipids in a membrane fraction enriched in the viral replication complex [30]. That study did not directly address the role of sphingolipids in DENV replication. In a different study, Wang et al. [47] exploited mouse melanoma WT cells (B16) and a mutant counterpart (GM95) to demonstrate that the glycosphingolipid GM3 is required for DENV genome replication. The authors found higher levels of GM3 in DENV-infected cells and relocalization of GM3 to sites where DENV replicates its genome. Importantly, the authors found that inhibition of GM3 synthesis with soyasaponin I increases the survival rate of DENV-infected mice [47]. While the exact role of GM3 in DENV genome replication is currently unknown, these in vivo findings raise the prospect that pharmacological inhibitors targeting GM3 synthesis can serve as a foundation for new antiviral therapy.

### 3.2. WNV Propagation and Sphingolipids

#### 3.2.1. Sphingolipids and WNV Entry and Genome Replication

The role of sphingomyelin in WNV replication is well documented. A study by Martin-Acebes and colleagues [48] showed that WNV replicates at a much higher level in mice deficient in acid sphingomyelinase (unable to catabolize sphingomyelin), or cells derived from Niemann–Pick disease type A patients (NPA; accumulate sphingomyelin) relative to the their wild type controls. This suggested that sphingomyelin accumulation enhances WNV infectivity. Consistent with these findings, adding sphingomyelin to infected fibroblast cells markedly increased WNV infectivity. Further analysis showed that sphingomyelin colocalizes with WNV dsRNA at cytoplasmic foci, implying that sphingomyelin plays a role in the formation of the WNV replication platform [48]. Interestingly, pharmacological inhibitors of sphingomyelin synthesis (DS609 and SPK-601) markedly reduced the infectivity of WNV released from infected cells, but had little impact on the amount of released viral genome [48]. These findings imply that sphingomyelin is also required for WNV attachment, internalization, and/or virus–endosome fusion.

#### 3.2.2. Sphingolipids and WNV Particle Formation

In an earlier report, Martin-Acebes and colleagues [49] also showed that WNV particles were enriched in sphingomyelin. Surprisingly, pharmacological inhibition of neutral sphingomyelinase (converts sphingomyelin into ceramide and phosphorylcholine) reduced WNV release from infected cells, implying perhaps that ceramide generated from sphingomyelin catabolism is critical for the infectious WNV particle. Subsequent analysis showed that inhibition of neutral sphingomyelinase activity reduces the budding of the immature WNV particles, a crucial step in infectious WNV particle formation [49]. While this study appears to be at odds with the putative role of sphingomyelin in WNV entry, it also highlights the role of sphingomyelin in two distinct steps of the WNV replication cycle.

### 3.3. ZIKV Replication and Sphingolipids

The role of sphingolipids and glycosphingolipids in ZIKV replication is not well understood. This is crucial because ZIKV patients with Guillain–Barré Syndrome, have elevated levels of antibodies targeting gangliosides GM2, GM1, GA1, and GD1 [27], hence highlighting the need to define the role of glucosylceramide-derived glycosphingolipids in ZIKV infectivity. Our group has evidence that ZIKV particles are enriched in sphingomyelin, ceramide, and glucosylecramide (unpublished data). Our current data suggest that glycosphingolipids leading to gangliosides biosynthesis (Figure 1) are required for ZIKV particle assembly (unpublished data). Current efforts are focused on understanding how sphingolipids and glycosphingolipids regulate ZIKV infectivity.

## 4. Human Immunodeficiency Virus’s Propagation and Sphingolipids

**Figure 4:**
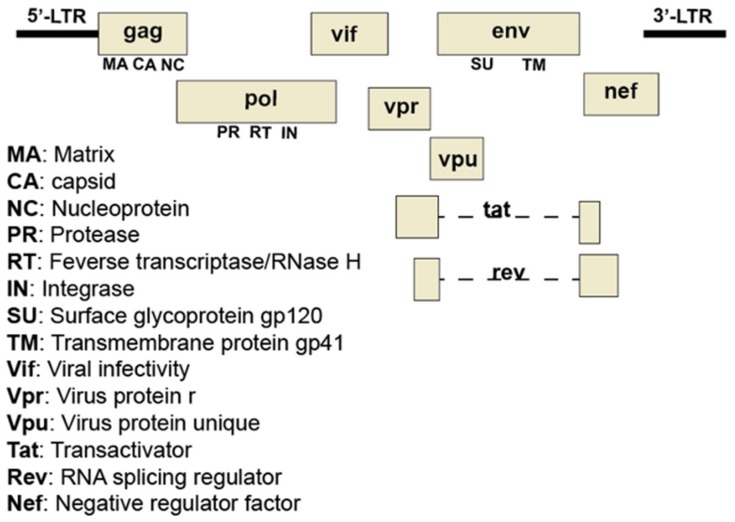
Organization of the HIV-1 genome. The HIV-1 genome consists of two identical copies of noncovalently linked, linear, positive-sense, single-stranded RNA molecules. Each identical copy contains nine genes that encode fifteen proteins. Many of the proteins are synthesized as precursor polyproteins which are proteolytically processed by host or viral proteases into individual proteins with roles in viral architecture, replication, regulation of cellular functions, and evasion of the host defenses. The gag gene encodes viral proteins involved in the structure of the virus. The pol gene encodes viral proteins critical for replication and integration of provirus into the host genome. The env gene encodes proteins needed for viral attachment and fusion with target cells.

Human immunodeficiency virus (HIV) is responsible for acquired immunodeficiency syndrome (AIDS) worldwide. In 2017 alone, approximately one-million people died of AIDS-related illnesses, highlighting the need for alternative or complementary remedies to eradicate HIV infection. HIV is an enveloped virus with two identical single-stranded RNAs which serve as a template for reverse transcription into a single double-stranded DNA intermediate that can integrate into the host genome. This DNA is the template for RNA that codes for new genomes or for viral proteins. Full-length RNA codes for a structural polyprotein that includes matrix (MA), capsid (CA), and nucleocapsid (NC) (Figure 4). Inefficient supression of a stop codon between the gag and the downstream pol reading frame produces a polyprotein that is extended to include the pol proteins protease (PR), reverse transcriptase (RT), and integrase (IN). All are cut into their constituent parts by PR. A singly spliced mRNA encodes the env glycoproteins gp41 (TM, transmembrane, a fusion protein) and gp120 (SU, surface glycoprotein). Other more extensively spliced messages, encode the regulatory proteins Tat, a transactivator of transcription, and Rev, an HIV-specific RNA exporter. Yet other messages code for accessory proteins Vif and Vpr, which facilitate the degradation of cellular defense proteins, and Vpu and Nef, which remove cellular proteins from the cell surface [50,51]. Knowledge of HIV biology and pathogenesis has lead to the identification of several pharmacological targets for antiretroviral drug therapy. Following approval of the first anti-HIV drug viral azidothymidine (AZT; nucleoside reverse transcriptase inhibitor), additional classes of drugs targeting viral attachment/fusion, viral genome replication, integrase activity, and the viral protease have been developed, some of which were subsequently included in combinational antiretroviral drug therapy, resulting in effective clinical management of HIV infection and AIDS-related illnesses [52]. Despite the efficacy and availability of these drugs, there exist several challenges that necessitate continued research in the identification of new viral targets and anti-HIV agents [53,54]. There is a continual threat of drug resistance due to HIV’s high mutation rate. Each class of currently available drug has the potential to cause acute and chronic toxicities in patients (e.g., cardiovascular and metabolic abnormalities). Lastly, side-effects of the drugs adversely impact patient compliance.

### 4.1. Glycosphingolipids and HIV Entry

Glycosphingolipids are also key players in HIV infection. HIV-1 infection of susceptible cells involves fusion of the HIV membrane with the host cell membrane. This process involves interactions between the viral gp120 and gp 41 envelope glycoproteins and CD4 and chemokine coreceptors (CXCR4 or CCR5) on the host cell. Several studies have implicated glycosphingolipids in the fusion process. Hug et al. demonstrated that glucosylceramide-derived glycosphingolipids found on the target cell membrane are involved in the organization of gp120–gp41, CD4, and chemokine receptors into a membrane fusion complex [55]. Rawat et al. reported that expression levels of the membrane ganglioside GM3 impact CD4-dependent viral fusion and infection [56]. Furthermore, several glycosphingolipids (galactosylceramide, GD3, Gb3, and GM3) have been reported to bind to the HIV gp120 envelope protein and contribute in some cases to the HIV infection of CD4 negative cells [57,58,59,60]. Specifically, HIV-1 entry was impaired in human colonic cells (HT29; CD4 negative cells) with a synthetic analog or a monoclonal antibody to galactosylceramide (GalCer) [60,61], implying that GalCer is an alternative receptor for HIV replication entry. Similarly, antibodies to GalCer reduced HIV infectivity andinternalization in two CD4-negative neural cell lines, U373-MG and SK-N-MC [58]. Altogether, these findings imply that glycosphingolipids contribute to HIV membrane fusion and can serve as alternative receptors for HIV entry in some cells.

### 4.2. Glycosphingolipids and HIV Budding

HIV particle assembly and budding takes place in the plasma membrane’s lipid rafts which contain high levels of cholesterol, sphingolipid (sphingomyelin), and glycosphingolipids. Notably, genetic analysis and lipid mass spectrometry showed that the HIV particle is enriched in sphingolipids (sphingomyelin and dihydro sphingomyelin) and glycosphingolipids (hexosylceramide, GM3, GM2, GM1) [62,63,64,65], implying that sphingolipids contribute to the biogenesis of infectious HIV particle and are acquired during HIV budding. There is also increasing evidence that glycosphinolipids help facilitate HIV’s infection of macrophages. Indeed, multiple studies have shown that GM3, GM2, and GM1 in HIV’s envelope interact with Siglec-1, a molecule that specifically binds to the sialyllactose moiety in glycosphinolipids [64,65,66]. This interaction between glycosphinolipids and Siglec-1 captures HIVs on macrophages, which might exist as a reservoir for HIV’s transmission to T cells.

## 5. Influenza Virus Propagation and Sphingolipids

**Figure 5:**
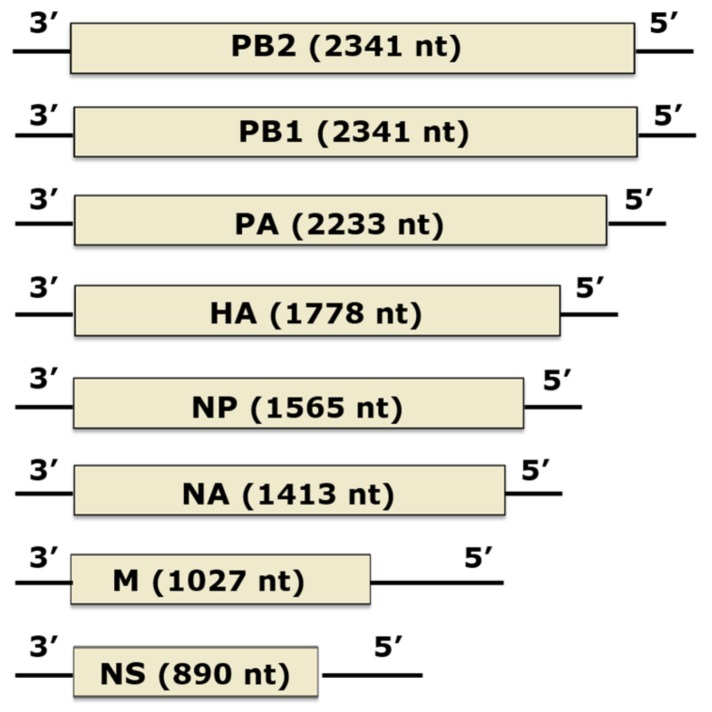
Organization of the Influenza A virus’s genome. The influenza A virus’s (IAV’s) genome consists of eight segments of negative-sense, single-stranded RNA. Encoded by the genome are three polymerase proteins (PA, PB1, and PB2), nucleoprotein (NP), and two envelope proteins (HA and NA). The M and NS mRNAs can be alternatively spliced to yield M1 and M2, and NS1 and NS2 proteins, respectively. Boxes indicate coding regions, sized relative to the other gene segments. Black lines at each end represent the 3’ and 5’ untranslated regions. The total length of each segment (conding and non-coding regions) in nucleotides (nt) is indicated. Figure adapted from Bouvier and Palese [71].

Seasonal influenza epidemics are responsible for over 200,000 hospitalizations in the United States and up to 500,000 deaths worldwide each year. Influenza A (IAV) is an enveloped virus possessing a negative sense, single-stranded, segmented RNA genome. Eight RNA segments encode 11 different viral proteins (Figure 5). The envelope spike glycoproteins HA and NA mediate viral entry and release, respectively, and serve as antigenic determinants of the virus. M2 is proton-selective ion channel involved in uncoating once the virus has entered the cell. Matrix protein (M1) forms a matrix under the viral envelope which is critical for maintaining the integrity and shape of the intact viral particle. Each segment of viral RNA is encapsulated with nucleocapsid protein (NP) and associated with the trimeric polymerase complex (PB1, PB2, and PA), forming what are referred to as a viral ribonucleoprotein complex (vRNP). Non-structural protein 1 (NS1) plays a critical role in evasion of host immunity. Non-structural protein 2 (NS2) is involved in the export of newly synthesized vRNPs from the nucleus to the cytoplasm for packaging.

The two classes of flu antiviral drugs include M2 ion channel blockers (e.g., amantadine and rimantadine) and neuraminidase inhibitors (e.g., zanamivir, oseltamivir). Early administration of these antivirals reduces disease symptoms, shortens the duration of illness, reduces hospitalization rates, and reduces viral transmission [67,68]. However, replication of the IAV genome involves a high error rate (10^−3^ to 10^−4^ substititution per genome) [69,70], resulting in the frequent accumulation of amino acid changes in IAV proteins. These changes enable IAV to evade host immunity acquired by prior exposure or vaccination and is the reason why IAV vaccines must be reformulated annually. Additionally, these amino acid changes may allow the virus to develop resistance against currently available antiviral agents that target the activity of the flu NA protein. As such, novel antivirals against influenza are critically needed.

### 5.1. Sphingolipids and IAV Entry

The influenza virus’s envelope, derived from the host cell’s plasma membrane, consists of a lipid bilayer decorated with the viral hemagglutinin (HA), neuraminidase (NA), and M2 proteins. The lipidome of purfied IAV particles consists of glycerophospholipids and sterols (primarily cholesterol) [72,73]. Further, almost all sphingolipid classes were detected in the viral envelope [72]. Attachment and entry into host cells requires interactions between the viral HA, concentrated in microdomains on the viral envelope, and sialic acid residues present on the cell surface. These microdomains, paralleling the lipids rafts present on the cell surface, are enriched with cholesterol and various sphingolipids, including sphingomyelin and glycosphingolipids [74,75,76]. Residues in the transmembrane domain and cytoplasmic tail of IAV HA are important for its association with lipid microdomains [75,76]. Disruption of these microdomains has an adverse effect on viral attachment. Sun and Whittaker showed that pretreatment of IAV virions with methly-β-cyclodextrin to deplete envelope cholesterol resulted in reduced viral fusion and infectivity [77]. Though not examined in their study, the authors suggested that cholesterol depletion perturbed the organization of HA in envelope lipid microdomains. Viral infectivity has been found to be reduced by approximately a thousandfold when HA fails to associate with lipid microdomains [75]. Similarly, it is expected that depletion of the IAV envelope sphingolipds would adversely impact IAV binding and infectivity.

### 5.2. Sphingolipids and IAV Replication

Following binding to host target cells, influenza virus enters the cytoplasm via receptor-mediated endocytosis. The viral M2 ion channel protein allows the influx of protons into the virion, triggering the release of viral ribonucleoproteins (vRNPs) into the cytoplasm. The vRNPs, made up of viral negative-stranded RNA, viral NP, and the viral RNA polymerase complex, then travel to the nucleus for transcription and replication of the influenza virus genome. Research suggests that products derived from sphingolipids are involved in influenza virus’s genome replication. Seo et al. [78] found that cells infected with influenza virus possessed increased levels of the enzyme sphingosine kinase (SK1), which converts sphingosine into sphingosine 1-phosphate (S1P). The authors further showed that inhibition of SK1 impaired viral RNA synthesis and the subsequent nuclear export of newly generated vRNPs. Similarly, it was demonstrated that SK1 is critical for the nuclear export of viral proteins (NP, NS2, and M1) involved in transporting vRNPs from the nucleus to the cytoplasm [79].

### 5.3. Sphingolipids and IAV Egress

Like several other enveloped viruses, influenza uses “raft-like” microdomains on the cell surface as platforms for viral assembly. Newly synthesized HA and NA concentrate in microdomains enriched for sphingomyelin and cholesterol [76,80,81,82]. Tafesse et al. [12] showed that perturbation of host sphingomyelin biosynthesis adversely impacted the trafficking of influenza virus HA and NA to the cell surface, which in turn impaired viral maturation, budding, and release. Additionally, though abbreviated treatment with methyl-β-cyclodextrin at the late stages of infection was found to increase the release of viral particles from infected MDCK cells, the infectivity of the released particles was significantly reduced, as their envelope possessed lower contents of cholesterol and disrupted raft microdomains [83].

## 6. Conclusions

**Figure 6:**
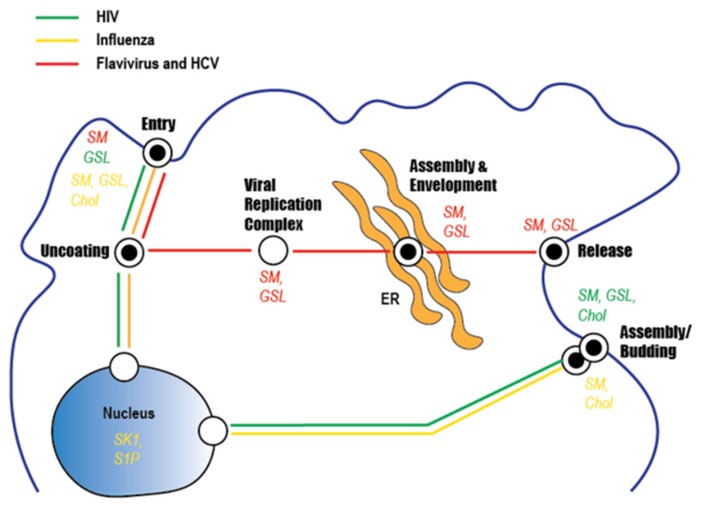
Sphingolipid involvement in the infection cycle of clinically important viruses. The three lines indicate the steps involved in the replication of HCV and flaviviruses (red), HIV (green), and influenza virus (yellow). Reported roles for lipids (sphingomyelin [SM], glycosphingolipids [GSL], and/or cholesterol (Chol)) in the lifecycle of each virus are indicated.

The role of sphingolipids and glycosphingolipids has been overlooked for many years in viral infection due to the difficulty of detecting or measuring these lipids. However, with the advent of lipid mass spectrometry, it is now possible to accurately determine the level of sphingolipids and glycosphingolipids in virus-infected cells and virus particles. Recent findings clearly imply that many enveloped RNA viruses have evolved to leverage sphingolipids and/or glycosphingolipids to enter target cells, replicate their genome, or form new virus particles enriched with these lipids (Figure 6).

Despite differences in the viral proteins and corresponding host cell receptor(s), the presence of sphingolipids in the envelopes of HCV, flaviviruses, HIV, and IAV appears critical for the proper organization of viral envelope proteins within microdomains to facilitate viral entry. Correspondingly, cell surface receptors required for viral adsorption and subsequent entry are concentrated in sphingolipid-rich microdomains present on host cell membranes, with sphingolipid itself serving as an alternative receptor for some viruses (e.g., HIV). Several of the viruses (e.g., HCV and DENV) discussed utilize specialized sites for genome replication. The trafficking of viral genomes and required replication platforms appear dependent on vesicular networks which involve lipid moieties, including sphingolipids. Lastly, the morphogeneses and egressions of the viruses discussed require the trafficking and assembly of components to budding sites on host membranes enriched for sphingolipids. Continued research is needed to ascertain the role of sphingolipids in the pathogenesis of these viruses and other enveloped RNA viruses.

Sphingolipids and glycosphingolipids are critical for membrane integrity and depleting them can have deleterious impact on distinct tissues or organs. However, minor changes in host glycosphingolipids can have a much greater impact on viruses that need them for successful infection. Hence, there is a need to develop more pharmacological inhibitors targeting sphingolipid and glycosphingolipid metabolic pathways. These inhibitors have the potential for a broad-spectrum antiviral activity. In addition, they can be combined with existing antivirals to increase their effectiveness and reduce the cost of these drugs.
